# Hippocampal beta rhythms as a bridge between sensory learning and memory-guided decision-making

**DOI:** 10.3389/fnsys.2023.1187272

**Published:** 2023-05-05

**Authors:** Jesse Thomas Miles, Kevan Scott Kidder, Sheri J. Y. Mizumori

**Affiliations:** ^1^Graduate Program in Neuroscience, University of Washington, Seattle, WA, United States; ^2^Department of Psychology, College of Arts and Sciences, University of Washington, Seattle, WA, United States

**Keywords:** beta oscillations, neural oscillations, decision-making, sensory learning, hippocampus

## Abstract

A pillar of systems neuroscience has been the study of neural oscillations. Research into these oscillations spans brain areas, species, and disciplines, giving us common ground for discussing typically disparate fields of neuroscience. In this review, we aim to strengthen the dialog between sensory systems research and learning and memory systems research by examining a 15–40 Hz oscillation known as the beta rhythm. Starting with foundational observations based largely in olfactory systems neuroscience, we review evidence suggesting beta-based activity may extend across sensory systems generally, as well as into the hippocampus and areas well known for coordinating decisions and memory-guided behaviors. After evaluating this work, we propose a framework wherein the hippocampal beta oscillation and its diverse coupling with other brain areas can support both sensory learning and memory-guided decision-making. Using this framework, we also propose circuitries that may support these processes, and experiments to test our hypothesis.

## Introduction

When researchers studying olfaction started recording from the hippocampus in the 1990s and 2000s they found that hippocampal activity could oscillate coherently with activity in early olfactory regions. The rhythmic coupling between these structures was strongest in the 15–40 Hz frequency band–the frequency of a classic olfactory system oscillation known as the beta rhythm. Prior work had indicated that beta rhythms occurred in other early sensory systems, such as the visual and auditory systems, and work since then has reaffirmed the existence of hippocampal beta in contexts that seemingly have nothing to do with active olfaction.

At the core of systems neuroscience is the promise of illuminating relationships between typically distinct sub-disciplines. Thus the goal of this review is to examine and relate the literature describing beta rhythms in sensory systems to beta rhythms in hippocampal processing. After briefly summarizing historical reports of beta, both in early sensory regions and the hippocampus, we will discuss new evidence suggesting cross-regional interactions between the hippocampus and a number of areas at beta frequencies. Based on these results, we will suggest that hippocampal beta is a distinctive rhythm that may have dual roles in sensory- and memory-guided behaviors.

Since this review will focus on sensory-cortical and hippocampal beta, as well as hippocampal beta coupling with other brain regions, we will not be reviewing all aspects of the beta rhythm. Instead, for reviews on beta rhythms in (primate) cortical information processing, we refer the reader to Spitzer and Haegens (2017) and Miller et al. (2018). To learn more about beta rhythms in motor systems, and how they become pathological in neurodegenerative diseases (see Stein and Bar-Gad, 2013; Singh, 2018; Barone and Rossiter, 2021). For general descriptions of how beta fits into circuit and system-wide oscillatory dynamics (see Kay et al., 2009; Kay, 2014; Kopell et al., 2014).

Before exploring the early work on beta, we should note that beta is not always defined in the same way, and its definition has changed over time. Fortunately, sensory systems work has been relatively consistent with its definition of beta as an often brief, 15–40 Hz rhythm. In contrast, early hippocampal research often separated rhythms into regular slow activity (often recognized as theta) and fast rhythms, which we would now call gamma, without consistent reference to their frequency content (Leung, 1992). To maintain consistency we will adopt the 15–40 Hz definition from the sensory systems literature in this review.

## Early reports of beta in sensory systems

Reports of beta rhythms in mammalian sensory systems extend at least as far back as the 1940s, when Adrian (1942) reported breathing- and scent-related 15–20 and 30–40 Hz rhythms in the hedgehog and rabbit olfactory bulb, hedgehog piriform cortex, and lateral olfactory tract of cats. Amplitude modulated sounds were shown to evoke 15–30 Hz responses in parts of the canine auditory cortex (Tielen et al., 1969), and recordings from the visual cortex of dogs trained to detect sinusoidally modulated light also showed beta oscillations (Lopes da Silva et al., 1970). As quantification of coordinated activity between neural systems became more precise, researchers began describing how different areas interacted with one another (Boudreau, 1964; Abraham et al., 1973; Holsheimer et al., 1979; Bressler, 1984; Boeijinga and Lopes da Silva, 1989; Wróbel et al., 1994). For example, Boeijinga and Lopes da Silva (1989) showed beta coherence between the piriform cortex and entorhinal cortex in cats exploring two different smells, especially as they sniffed an odor associated with reward. Others suggested that beta activity in the visual cortex during attention to visual stimuli propagated to the lateral geniculate nucleus (Wróbel et al., 1994). It’s unclear if there was consensus about general roles of the beta observed throughout sensory cortical areas. However, an attempt to summarize the role of beta oscillations specifically in visual processing suggested that inter-areal beta activity was a marker of attention, and posited that the same could be true of any sensory processing areas (Wróbel, 2000).

## Early reports of hippocampal beta

Explicit observations of a hippocampal beta rhythm in early research were scarce, but there were several notable exceptions. First, Boudreau showed hippocampal auto- and cross- spectral peaks in the 15–20 Hz range of awake cats (Boudreau, 1966). Comparing theta and beta generation in the hippocampus and overlying neocortex of the rat, one group concluded that, while there were many similarities between hippocampal theta and beta, they also appeared independently of one another (Holsheimer et al., 1979). Soon after, a report in awake rats proposed three main hippocampal rhythmic states–theta or regular slow activity, irregular slow activity, and fast waves. While this research described irregular slow activity as occurring partially in the beta range, it also attributed much of the energy in the beta range to theta harmonics (Leung et al., 1982), which is somewhat at odds with the reports of Holsheimer et al. (1979). In sum, early work on hippocampal beta described its similarities to theta, but suggested there were grounds to think of it as a separate rhythm.

## Beta coupling between the hippocampus and sensory cortices

A series of papers in the 1990s helped bridge the gap between sensory systems and hippocampal work by studying hippocampal and olfactory bulb processing in tandem. Hippocampal dentate gyrus recordings showed that 15–40 Hz “fast waves” were triggered when rats were coaxed to sniff toluene, xylene, or several predator-mimicking odors (Vanderwolf, 1992; Heale et al., 1994). Though not quantified, the authors also claimed the dentate fast wave was not necessarily correlated with olfactory bulb beta. Similar results were also obtained by Chapman et al. (1998), who also showed beta in the entorhinal and piriform cortices. Other researchers described a series of bidirectional interactions between the dentate gyrus, olfactory bulb, entorhinal cortex, and piriform cortex throughout the course of an odor discrimination (Kay et al., 1996; Kay and Freeman, 1998). With respect to beta, they described a so-called “preafferent” beta signal, originating in the entorhinal cortex and sent to the olfactory bulb prior to olfactory stimulus presentation. Overall, these authors suggested that beta in limbic structures could bias attention in early olfactory areas to detect learned stimuli, but it was still hard to say how the hippocampus factored into this process.

Following up on these results, Martin et al. (2007) recorded from the olfactory bulb and dentate gyrus while rats learned to distinguish between different pairs of odors. They found increased beta power in both structures during odor sampling, but beta coherence reliably increased only when rats learned the distinction between new odor pairs. Re-examination of these results with different mathematical techniques suggested that beta coherence flowed from the olfactory bulb to the hippocampus during odor sampling (Gourévitch et al., 2010). Looking deeper into the link between intra-limbic beta activity during odor identification, Igarashi et al. (2014) showed strong coherence between the lateral entorhinal cortex and dorsal CA1 region of the hippocampus during odor sampling. These authors also showed that learning was accompanied by increased beta coherence between these areas, which coincided with the formation of odor representations in cell populations. Further, they showed that error trials and changes of odor contingencies were accompanied by reduced coherence and reduced ensemble selectivity between areas. Together, these reports suggested that beta during sensory sampling can, but does not necessarily, coincide with beta in limbic structures, while increased entorhinal-hippocampal beta coupling tracks learning and task performance.

Building on olfactory-hippocampal beta coupling and early sensory systems work, one recent study demonstrated beta-based hippocampal interactions with non-olfactory sensory areas. To clarify how rhythmic activity was patterned across sensory cortices, Vinck et al. (2016) recorded from a primary somatosensory area (barrel cortex), primary visual cortex, perirhinal cortex, and dorsal CA1 of rat hippocampus. The strongest coupling between areas was in the beta range. This was different from local synchrony measures, which were strongest in the gamma range. Beta coupling was also stronger while animals were moving, but the authors did not study how rhythms correlated with other aspects of behavior. This study provided new evidence that the hippocampus could couple with a variety of cortical sensory regions in the beta frequency range, not just olfactory and higher-order limbic regions like the entorhinal cortex.

**FIGURE 1:**
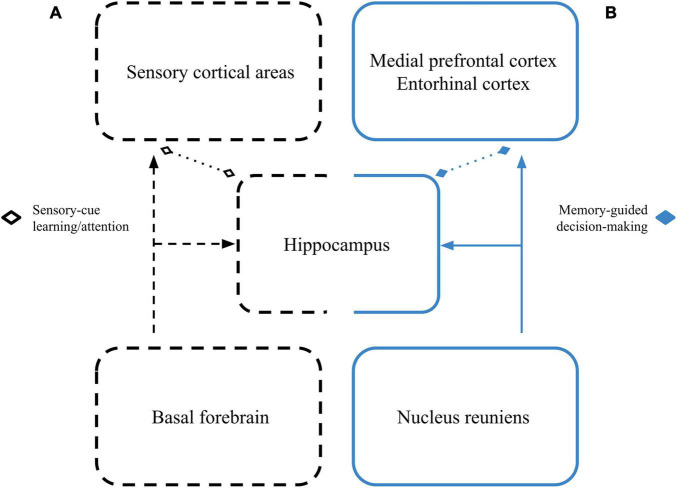
Dual roles for hippocampal beta networks. **(A)** Cues driving beta in sensory cortical areas may become coherent with cue-driven hippocampal beta through beta-related activity of cell assemblies in the basal forebrain as learning progresses and cues are selectively attended to. This network is represented by the black dashed boxes. **(B)** During memory-guided decision-making, the reuniens may coordinate beta between the hippocampus, prefrontal/association cortices, and their efferent action selection areas. This network is represented by the solid blue boxes. Dotted lines with diamond tips signify that these beta-based interactions may happen with or without direct anatomical connectivity.

Though it’s not yet clear how beta between the hippocampus and sensory cortices becomes coordinated, one possible candidate is through interactions with the basal forebrain (Figure 1). The basal forebrain has modular anatomical connections throughout the neocortex and with the hippocampus (Woolf, 1991; Záborszky et al., 2018). Further, beta-rhythmic basal forebrain local field potential (LFP) has been reported to change throughout learning (Quinn et al., 2010), and both cell assembly formation and oscillatory dynamics in the basal forebrain have been shown to occur at beta-rhythmic frequencies throughout the course of a trial in complex sensory-motor tasks (Tingley et al., 2015, 2018).

## Hippocampal beta in learning and memory

Recent work has provided more details on how hippocampal beta relates to learning and memory. Using a task that combined sensory-guided behavior with sequence-memory, Allen et al. (2016) showed the magnitude of rhythmic hippocampal activity in the 20–40 Hz range was stronger when odors were presented in a correctly learned sequence. Additionally, the magnitude increase correlated with task performance. Importantly, because they presented the same odors for correct and incorrect sequences, the authors argued that changes in beta were tied to the sequences themselves and not sensory aspects of the odor identities. Similar results in the same paradigm further demonstrated that this elevated hippocampal beta response tends to occur late in the odor-sampling process, particularly for odors correctly identified as being presented in the correct sequence (Gattas et al., 2022).

Interested in characterizing the relationship between hippocampal cell classes and LFP dynamics with respect to behavior, Rangel et al. (2016) recorded from dorsal CA1 during an odor-place association task. Similar to Allen et al. (2016), they found that beta-rhythmic activity was most strongly related to successful task performance. Specifically, the vast majority of putative interneurons and principal cells that phase-locked to the hippocampal LFP in the beta range did so only when animals chose correctly. Notably, beta-coherent principal cells were the only cells to selectively carry information about odor-place associations, and only during the period coinciding with their decision. From these data, the authors concluded that beta-rhythmic activity might be uniquely situated to process information required for memory-guided associations. This mirrors other findings that hippocampal LFP beta power increased in response to reward-predictive cues, concurrent with decreased theta, in a variety of cue-reward association tasks (Rangel et al., 2015).

While most research on hippocampal beta has come from studies using olfactory stimuli, Berke et al. (2008) demonstrated dorsal hippocampal beta power increasing in response to novel environments. These authors showed that strong beta oscillations in CA1 and CA3 emerged early in sessions where mice had been introduced to novel environments, with spiking phase-locked to the beta LFP oscillation, and spatial specificity in place cells emerging during the elevated beta period. These results provided evidence that the hippocampus could exhibit beta-rhythmic activity during behaviors that were not explicitly sensory-guided. In sum, work focused on hippocampal beta has shown that its prevalence extends beyond a simple role in olfactory learning by linking it with novelty (Berke et al., 2008), sequence memory (Allen et al., 2016; Gattas et al., 2022), and cue-reward associations (Rangel et al., 2015, 2016).

## Beta coupling between the hippocampus and “non-sensory” areas

Showing that beta oscillations could link the hippocampus and non-sensory areas, Lansink et al. (2016) reported increased beta (and theta) activity between the hippocampus and ventral striatum during cue-driven navigation. Similar to Rangel et al. (2015), they showed that entry into a cued location associated with reward caused increases in hippocampal beta/theta LFP power and intra-hippocampal coherence. Additionally, spike timing in the ventral striatum showed beta-rhythmic phase-locking to the dorsal CA1 LFP, which, again, was stronger when the animal approached a cued reward. Therefore, the authors suggested that beta and theta rhythmic interactions between the dorsal hippocampus and ventral striatum were important when anticipating reward and guiding behavior based on learned associations to cues.

Although the hippocampus and medial prefrontal cortex (mPFC) are classically known for their theta oscillatory coupling (Jones and Wilson, 2005; Siapas et al., 2005; Benchenane et al., 2010; Hyman et al., 2010; Colgin, 2011; Gordon, 2011), recent studies have shown beta-based interactions between the two. In an odor-place association task, beta coherence between the mPFC and hippocampus increased as rats were sampling odors and making decisions about where they would navigate (Symanski et al., 2022). Cells that phase-locked to the local beta rhythm in either mPFC or hippocampus did so more strongly before correct decisions, but no clear differences in LFP coherence between areas was observed. It’s worth noting that these authors also saw beta coherence between both structures (mPFC and hippocampus) and the olfactory bulb during the same odor sampling and decision-making period, suggesting that this network is engaged during active sensation at beta frequency as well. This is somewhat at odds with prior reports showing only occasional coherence between the olfactory bulb and hippocampus during odor sampling, but in accordance with suggestions that different behaviors and behavioral strategies may alter beta dynamics (Gourévitch et al., 2010; Kay and Beshel, 2010; Frederick et al., 2016).

In another study using the previously mentioned olfactory sequence-memory task, Jayachandran et al. (2022) found that hippocampal beta during sequence-memory is coherent with mPFC beta. Accurate identification of correctly ordered sequences showed higher beta coherence than sequences that were incorrectly ordered or misidentified. Interestingly, these authors also found beta bursts in recordings from the reuniens that began just prior to beta in the mPFC and hippocampus, and showed that optogenetic stimulation of reuniens projections to the hippocampus caused beta in the hippocampus and mPFC.

Taken together, these results show that coordinated beta-rhythmic activity can exist between the hippocampus and areas not traditionally considered sensory processing regions, such as the mPFC and ventral striatum. Beta coupling between the hippocampus and mPFC seems to be linked to successful memory-based decision-making (Igarashi et al., 2014; Jayachandran et al., 2022; Symanski et al., 2022), beta-rhythmic activity between the ventral striatum and hippocampus is strongest during cue-triggered reward expectation (Lansink et al., 2016), and hippocampal-entorhinal beta seems to track learning (Igarashi et al., 2014). All of these results suggest that increased beta between the hippocampus and higher-order cortical or action planning areas is important for successful memory-guided behavior.

## Dual roles for hippocampal beta networks

What remains unclear is whether beta-based interactions between the hippocampus and sensory areas have the same characteristics as beta-based interactions between the hippocampus and “non-sensory” areas (Figure 1). The olfactory system, for example, seems to reliably exhibit beta oscillations during active sensation, but that activity is not always coherent with hippocampal beta activity (Kay and Freeman, 1998; Martin et al., 2007; Gourévitch et al., 2010), even when animals are engaging in a learned behavior. On the other hand, there are increases in beta coherence between the hippocampus and olfactory sensory areas during rule transfers to new stimuli (Martin et al., 2007; Gourévitch et al., 2010), and freely moving rats can show beta-rhythmic activity between the hippocampus and a variety of sensory areas even if they are not engaged in any structured task (Vinck et al., 2016).

Resolving this ambiguity will require more studies that record concurrently from the hippocampus and sensory areas during active sensation, as has been done in the olfactory system. Analogous tests of the visual system could be done using tasks that change which visual cues are associated with reward. Presumably, visual cortex and hippocampus would show beta during cue presentations, but coherence between areas would only significantly increase while learning cue-reward associations or after they change. The test could be made even stronger using a task that switched reward contingencies between different modalities of sensory cue (e.g., visual and olfactory/auditory). If beta coherence between the hippocampus and sensory structures switched based on rewarded sensory modality, it would support the idea that beta coherence enables sensory-driven, cue-reward association. Additionally, if the cross-regional coherence increases were specific to transition periods, it would suggest that learned sensory cues *per se* do not drive the interaction, but the flexible contingency re-learning or attentional shift required to update behavior does.

We also have reason to believe that hippocampal beta coupling with non-sensory areas has relevance for task performance, learning, and memory (Igarashi et al., 2014; Lansink et al., 2016; Jayachandran et al., 2022; Symanski et al., 2022). The tasks from these studies, however, are all explicitly tied to sensory stimuli, and it’s clear that hippocampal beta can occur under conditions not specifically locked to reward (Berke et al., 2008; Vinck et al., 2016). Recordings from hippocampus and areas linked to higher-order association and decision-making during tasks that do not require sensory-driven behavioral responses would help clarify how beta coupling unfolds between hippocampus and non-sensory areas. For example, spatial working memory tasks often require hippocampal-prefrontal interactions (Floresco et al., 1997; Wang and Cai, 2006; Eichenbaum, 2017), but there do not seem to be experiments directly asking whether beta-based activity correlates with spatial working memory or decision-making. We would expect to see brief elevations in hippocampal-prefrontal beta coherence as decisions are made about where to navigate. Care should also be taken to ensure the task requires allocentric navigation. This would prevent simple cue-based strategy use, which we already suspect causes hippocampal beta.

## Conclusion

We hypothesize that one role for hippocampal-based beta is to coherently oscillate with sensory areas and promote cue-reward associations, while another is to coherently oscillate with decision-making and action selection areas, enabling successful memory-guided behavior. Continued study of neural coordination within and across sensory and memory systems could reveal new insights into the nature and significance of beta-based activity across disparate brain structures (Kopell et al., 2000).

## Author contributions

JM, KK, and SM contributed to the conceptualization of the review. JM wrote the initial drafts of the document, proposed the model, and made the associated figure. All authors contributed to manuscript revision, read, and approved the submitted version.
